# Incidental Primary Ovarian Squamous Cell Carcinoma Arising in Endometriosis Presenting As Adenomyosis-Related Abnormal Uterine Bleeding

**DOI:** 10.7759/cureus.90715

**Published:** 2025-08-22

**Authors:** Kavita Mardi, Kanav Goyal, Arnav Jagota

**Affiliations:** 1 Pathology, Indira Gandhi Medical College, Shimla, IND

**Keywords:** abnormal uterine bleeding, adenomyosis, adjuvant chemotherapy, debulking surgery, endometriosis, histopathology, ovarian squamous cell carcinoma

## Abstract

Adenomyosis often coexists with endometriosis, which primarily involves the ovary and can lead to endometriosis‑associated ovarian cancers, most commonly endometrioid and clear‑cell carcinomas. Ovarian squamous cell carcinoma (OSCC) arising in endometriosis is exceedingly rare, lacks established treatment guidelines, and carries a poor prognosis despite only sporadic reports in the literature. We describe a 45‑year‑old multiparous woman with a three‑month history of abnormal uterine bleeding. Speculum examination revealed a bleeding polypoid cervical mass, and transvaginal ultrasound confirmed adenomyosis. Persistent bleeding refractory to polypectomy and resultant severe anemia prompted total abdominal hysterectomy with bilateral salpingo‑oophorectomy. Unexpected histopathology and immunohistochemistry revealed a primary, poorly differentiated OSCC of the left ovary arising from endometriosis, extending into the left fallopian tube; the uterus, cervix, right adnexa, and ovarian capsule were uninvolved. The tumor was staged International Federation of Gynecology and Obstetrics IIA, and the patient received six cycles of carboplatin plus paclitaxel. At 15 months postoperatively, she remains disease‑free. This case underscores how adenomyosis can mask an underlying ovarian malignancy and illustrates the rare occurrence of OSCC in endometriosis. Awareness of such presentations should prompt thorough histological evaluation, and we emphasize the urgent need to develop treatment protocols for rare, aggressive histotypes. We also review the existing literature to contextualize this uncommon pathology and its clinical implications.

## Introduction

Adenomyosis is a benign uterine disorder in which ectopic endometrial glands and stroma invade the myometrium, accompanied by smooth muscle hyperplasia. It commonly presents with abnormal uterine bleeding (AUB), pelvic pain, dysmenorrhea, dyspareunia, or infertility, although one‑third of patients are asymptomatic. Risk factors include age over 40 years, multiparity, prior cesarean delivery, and other uterine surgeries; it is increasingly diagnosed in younger women with infertility, pelvic pain, or AUB [1]. Transvaginal ultrasound is the first‑line diagnostic tool, with key findings including asymmetrical uterine wall thickening, intramyometrial cysts or hyperechoic islands, fan‑shaped myometrial shadowing, subendometrial echogenic lines and buds, translesional vascularity, and an irregular junctional zone. Histology of hysterectomy specimens remains the gold standard, but improved imaging now allows preoperative identification [1]. Treatment is tailored to age, reproductive goals, and symptoms: conservative options (e.g., endometrial ablation, hysteroscopic resection, laparoscopic adenomyoma excision, high‑intensity focused ultrasound, uterine artery embolization) are preferred in women desiring fertility, whereas hysterectomy is definitive for those with completed childbearing or concomitant pathology [1]. Adenomyosis frequently coexists with endometriosis and fibroids; leiomyomas coexist in 15-57% of cases and exacerbate pelvic pain. Historically termed “endometriosis interna” due to its overlap with endometriosis, adenomyosis has a prevalence of 20-80% among women with endometriosis [1].

Endometriosis is an inflammatory condition characterized by functional endometrial tissue outside the uterine cavity. It affects 5-10% of reproductive‑age women and 2-4% of postmenopausal women. Symptoms include dysmenorrhea, chronic pelvic pain, dyspareunia, dyschezia, infertility, vaginal bleeding, or ovarian masses; some women remain asymptomatic. Diagnosis requires clinical evaluation, imaging, laparoscopy, and histology [2]. Although benign, endometriosis carries a 1-2% risk of malignant transformation. When this occurs, about 80% of cases develop endometriosis‑associated ovarian cancers (EAOCs), but rare extraovarian transformations have also been reported in the abdominal wall, rectovaginal septum, and intestine. EAOCs most often arise in perimenopausal women (mean age 55.8 ± 8.6 years), with risk increasing alongside age, hyperestrogenism, and larger endometrioma size (≥9 cm). Endometrioid and clear‑cell ovarian carcinomas are the EAOCs most frequently encountered, whereas primary ovarian squamous cell carcinoma (OSCC) arising in endometriosis is exceptionally rare [2].

Ovarian cancer is the eighth most common malignancy in women worldwide, encompassing a broad spectrum of histological subtypes [2]. The most prevalent are epithelial tumors, including serous, mucinous, endometrioid, clear-cell, Brenner, and squamous cell carcinomas. Meanwhile, other subtypes include sex cord-stromal tumors and germ cell tumors such as teratomas [2,3]. Primary OSCC is exceedingly rare, accounting for less than 1% of all ovarian cancers [3-6]. It most commonly arises from malignant transformation of mature cystic teratomas [6], less frequently from squamous metaplasia within endometriosis [3,7-9] or Brenner tumors [10], and very rarely occurs as pure primary OSCC arising from metaplasia of the ovarian surface epithelium without any preexisting lesion [5]. Clinical presentation varies with disease stage: early-stage disease may cause vague pelvic or abdominal tenderness, while advanced stages may present with abdominal distension, palpable masses, pain, vaginal bleeding, bowel or bladder dysfunction, and systemic symptoms such as weight loss [6]. Preoperative diagnosis is challenging, as clinical signs, tumor markers, and imaging lack specificity; many early-stage tumors are identified only incidentally on postoperative histopathological analysis [6].

EAOCs are managed surgically, followed by platinum‑ and taxane‑based chemotherapy, following epithelial ovarian cancer treatment protocols [2]. However, due to its rarity and aggressive behavior, OSCC lacks standardized treatment guidelines and is typically managed based on published case reports. Recommended therapy entails cytoreductive surgery, including bilateral salpingo‑oophorectomy with or without hysterectomy, lymphadenectomy, and omentectomy, followed by adjuvant carboplatin plus paclitaxel chemotherapy. Despite these interventions, OSCC is associated with poor prognosis and reduced survival [3-10].

McCullough et al. first reported primary OSCC associated with endometriosis in 1946, followed by Lele et al. In 1978 [3,9]. Fewer than 30 well‑documented cases have since been reported, generally associated with poor prognoses [3,7,8]. Adding to the limited literature, we present an incidental case of primary, poorly differentiated OSCC arising from endometriosis in a 45‑year‑old multiparous woman, whose clinical presentation was dominated by heavy bleeding due to adenomyosis. This case underscores the importance of comprehensive histopathological evaluation and lays the groundwork for future treatment guidelines combining optimal surgery and adjuvant chemotherapy.

## Case presentation

A 45‑year‑old multiparous woman presented to our tertiary hospital in Shimla, India, in March 2024 with a three‑month history of AUB. Her menstrual cycles had been regular, lasting three to four days every 28 to 32 days with moderate flow (three to four pads per day), until three months prior to presentation. At that time, she developed continuous heavy bleeding, with menses lasting 15-20 days and requiring eight to ten pads per day, frequently accompanied by passage of clots. During the preceding ten days, she experienced progressive dyspnea on exertion, fatigue, and dizziness. She also reported dyspareunia and significant weight loss over the same three-month period. She denied hormone replacement therapy, abdominal pain or distension, abnormal vaginal discharge, post‑coital bleeding, or protruding masses, and had no relevant family history. Her obstetric history included three full-term vaginal deliveries and a laparoscopic bilateral tubal ligation 10 years earlier. On examination, she appeared pale but had no clinical lymphadenopathies or ascites. Speculum inspection revealed minimal bleeding through the cervical os and a 3 × 1 cm polypoid mass. Her hemoglobin was 5.2 g/dL. Pelvic ultrasound showed a bulky, heterogeneously echogenic uterus with a poorly defined junctional zone, 9.8 mm endometrial thickness, and a posterior myometrial lesion suggestive of adenomyosis or fibroid. Both adnexa appeared grossly normal, and there was a small amount of free fluid in the pouch of Douglas.

**Figure 1:**
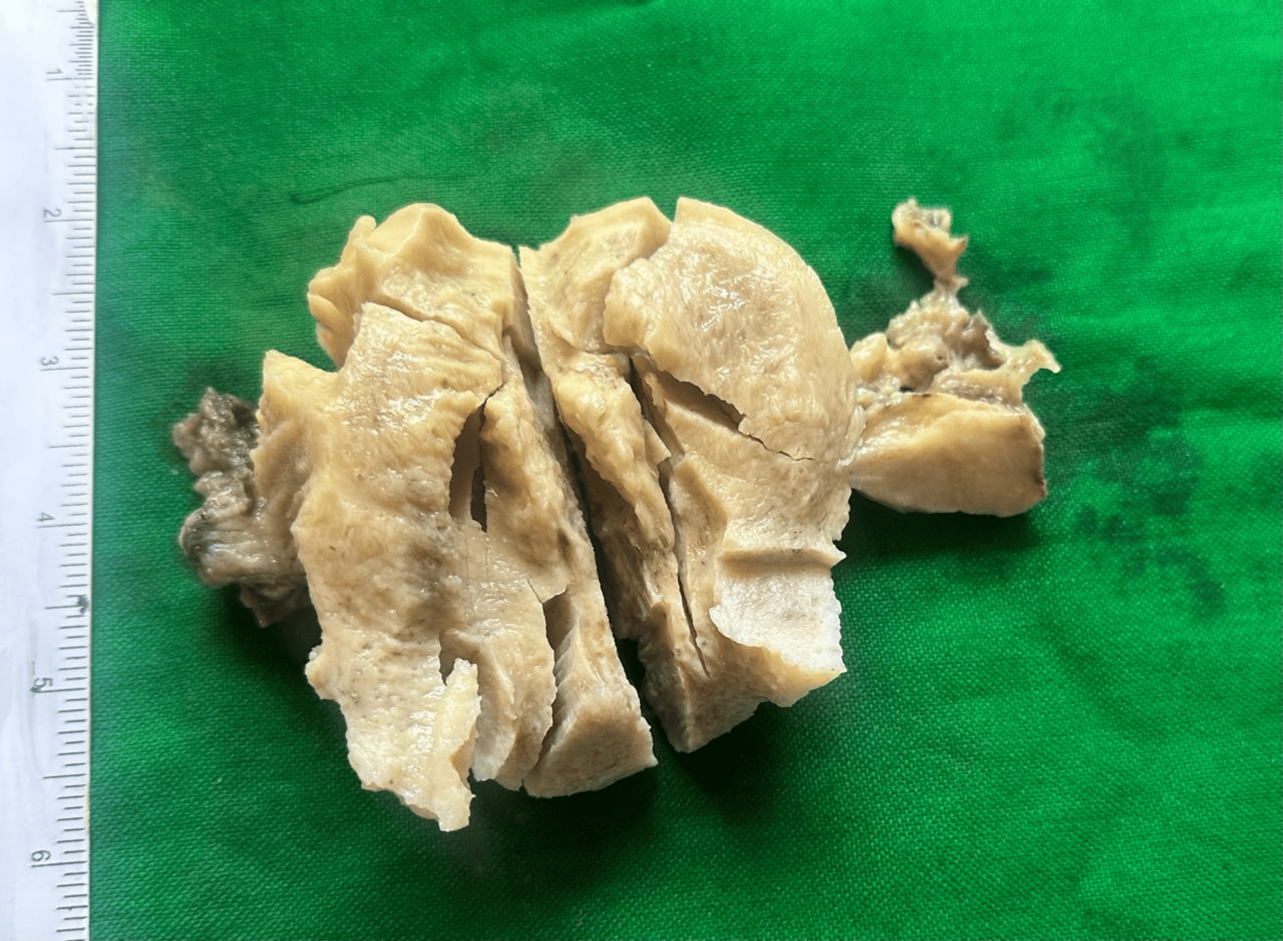
Gross specimen of uterus with cervix, bilateral fallopian tubes, and ovaries showing hypertrophied cervix and solid left ovary

Despite polypectomy, hemorrhage persisted, and her hemoglobin fell to 3.0 g/dL. Given her life-threatening anemia, informed consent was obtained, and a laparoscopic hysterectomy was planned. Intraoperatively, a left tubo-ovarian mass was found to be adherent to both bowel and bladder, and an endometrial growth measuring 3 × 4 cm was noted on the posterior uterine wall with dense adhesions between the posterior uterine wall and the rectosigmoid colon. Because of these unexpected findings, the procedure was converted to an open total abdominal hysterectomy with bilateral salpingo-oophorectomy and exploration of the abdomen and pelvis. The remainder of the pelvis, including the lateral pelvic walls and pelvic floor, appeared grossly normal, and no other intra-abdominal abnormality was identified. Peritoneal washings and omental biopsies were obtained. Two units of packed red blood cells were transfused intraoperatively. The surgical specimen comprised the uterus with cervix (8 × 7 × 3 cm), bilateral fallopian tubes (approximately 4 cm each), and ovaries measuring 3 × 1.5 × 0.5 cm (right) and 4 × 3 × 0.5 cm (left). On the cut section, the endometrium measured 1-2 mm and the myometrium 2 cm. The cervix was hypertrophied and everted. The left ovary appeared solid and gray-white; the right ovary was unremarkable (Figure 1).

**Figure 2:**
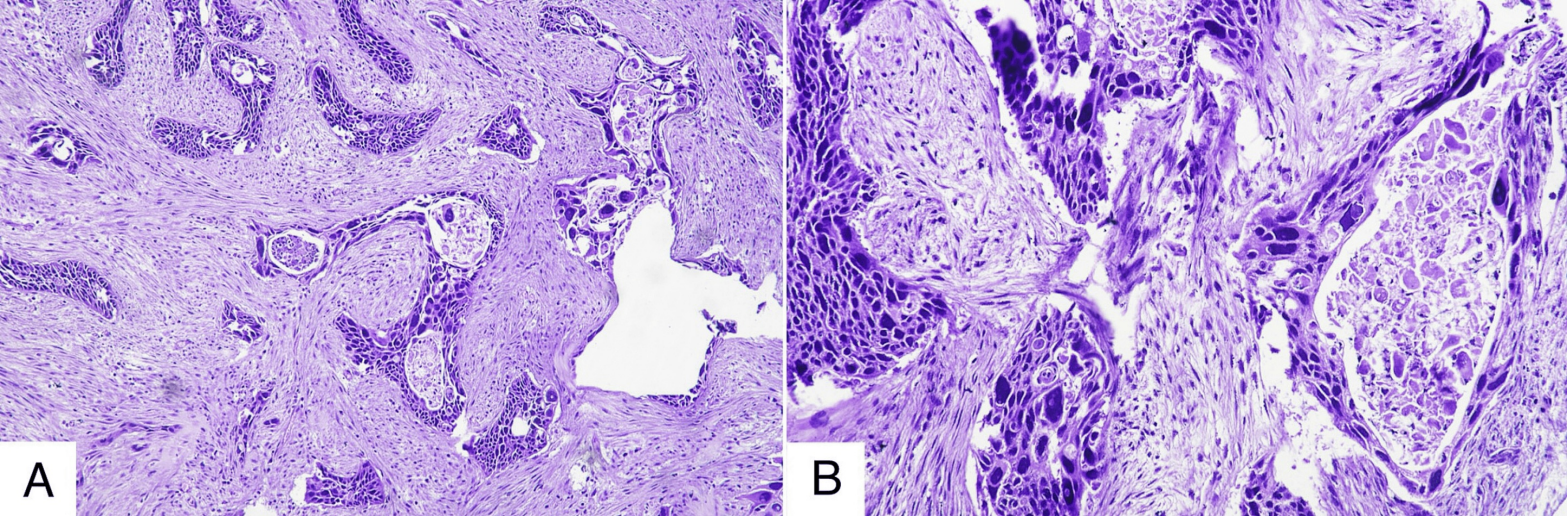
Histopathological evaluation of the left ovary demonstrating pleomorphic tumor cells in nests and cords with desmoplastic stroma, acantholysis, dyskeratosis, and tumor necrosis A. Lower magnification. B. Higher magnification.

On microscopic examination, the endometrium was in the weak proliferative phase, and the endometrial polyp displayed benign features. The myometrium showed adenomyosis, while the cervix and right fallopian tube exhibited chronic non-specific cervicitis and salpingitis, respectively. The right ovary was histologically unremarkable. In contrast, the left ovary was replaced by markedly pleomorphic tumor cells forming variably sized nests, trabeculae, cords, and isolated cells within a dense desmoplastic stroma. Pseudoglandular areas with central acantholytic cells were noted in the center of the nests. The neoplastic cells had irregular, elongated, hyperchromatic nuclei, inconspicuous nucleoli, and moderate to abundant eosinophilic cytoplasm with focal dyskeratosis. Atypical mitotic figures, numerous tumor giant cells, and frequent nuclear pseudoinclusions were observed. Many nests exhibited central necrosis and apoptotic bodies, with additional separate foci of tumor necrosis. No normal ovarian parenchyma could be identified, and the ovarian capsule remained intact; there was no lymphovascular or perineural invasion (Figure 2A-2B).

**Figure 3:**
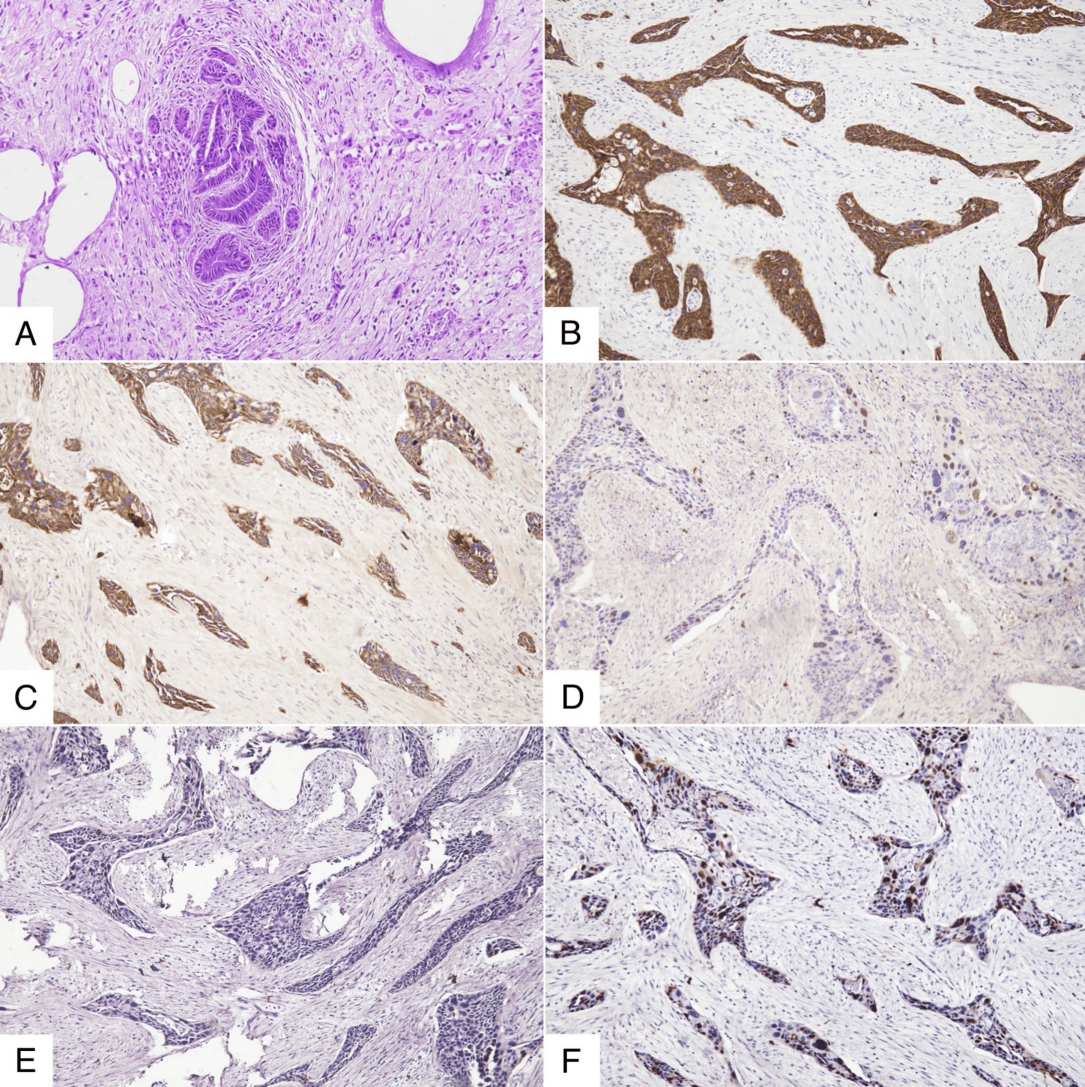
Histopathology and immunohistochemistry showing endometriosis foci, CK5/6, CK7, focal p40 positivity, p16 negativity, and a Ki-67 index of 70% A. Section of the left ovary showing foci of endometriosis. B. CK5/6 (cytokeratin 5/6) diffusely positive. C. CK7 (cytokeratin 7) diffusely positive. D. p40 focally positive. E. p16 negative. F. Ki-67 (marker of proliferation Kiel 67) index of 70%.

Tumor infiltration extended into the left fallopian tube, and several ovarian sections demonstrated areas of endometriosis (Figure 3A). No Brenner tumor or mature cystic teratoma was present, and the cervix showed no evidence of squamous cell carcinoma. Immunohistochemistry showed diffuse positivity for CK5/6 (cytokeratin 5/6) and CK7 (cytokeratin 7), along with focal positivity for p40 (Figure 3B-3D). It also showed negativity for p16 and a high Ki‑67 (marker of proliferation Ki-67) proliferation index of 70% (Figure 3E-3F). Histopathological and immunohistochemical findings support a diagnosis of primary, poorly differentiated OSCC of the left ovary arising in endometriosis, with extension into the left fallopian tube. The uterus, cervix, right adnexa, and right fallopian tube were free of tumor. Cytology of the peritoneal washings and histopathology of the omental biopsies showed no evidence of metastasis. Ultrasonography and CT scans showed no lymph node involvement or distant metastasis. The disease was staged as International Federation of Gynecology and Obstetrics (FIGO) IIA.

She received six cycles of carboplatin plus paclitaxel every three weeks with good tolerance. At 15 months postoperatively, she continues three‑monthly follow‑up, remains disease‑free, and has returned to normal activities.

## Discussion

Endometriosis is not a pre-cancerous condition but a benign disease with malignant potential, carrying a 1-2% risk of malignant transformation, most often to EAOC [2]. Epithelial ovarian cancers arise when extraovarian cells implant on the ovary and undergo malignant transformation: fallopian-tube epithelium gives rise to serous carcinoma; transitional epithelium from the tubo-peritoneal junction, to mucinous carcinoma; and endometriotic implants, to endometrioid, clear-cell, or very rarely OSCC [3]. Primary OSCC is exceedingly rare (<1% of ovarian cancers) and most often emerges from malignant transformation of mature cystic teratomas; less commonly, it originates in endometriosis via squamous metaplasia, in Brenner tumors, or, in a handful of reports, from metaplasia of the ovarian surface epithelium without antecedent lesions [3-10]. Secondary OSCC represents metastasis from other organs, and because squamous cell carcinoma commonly arises in the cervix, vulva, lung, head, and neck, thorough evaluation, including physical examination, cytology, and imaging, is required to exclude metastases before diagnosing primary OSCC [5]. Preoperative diagnosis is challenging because symptoms, tumor markers, and imaging lack specificity, so many OSCCs are only identified incidentally on postoperative pathology [6]. Histologically, OSCC shows keratinizing squamous nests, and immunohistochemistry shows CK5/6 and p40 positivity [7]. Human papillomavirus (HPV) is a common oncogenic driver in OSCC, often presenting as synchronous cervical and ovarian tumors [6]. Negativity for p16 helps exclude the possibility of HPV-related tumors, which are generally secondary. A high Ki-67 index reflects aggressive behavior, as seen here. Serology may show elevated CA125 (cancer antigen 125) and CA19-9 (cancer antigen 19-9) [3]; we did not perform these tests because malignancy was not suspected preoperatively. Extensive sampling is essential, as the tumor can overgrow its origin, often obscuring endometriotic foci, making identification of the primary lesion challenging [4]. Given its rarity, the literature on OSCC arising in endometriosis is confined to case reports and small series [3,7-9].

Adenomyosis may coexist with other uterine pathologies, complicating the clinical picture [1]. OSCC arising in endometriosis typically presents in perimenopausal women with nonspecific symptoms such as abdominal or pelvic pain, distension, palpable mass, bleeding, or symptoms related to metastasis [2]. In contrast, our patient’s presentation with life-threatening AUB due to presumed adenomyosis is unusual. Histopathological evaluation of the hysterectomy specimen incidentally revealed primary OSCC arising from endometriosis. Per speculum examination had shown uterine polyps, and imaging was consistent with adenomyosis. Total abdominal hysterectomy with bilateral salpingo-oophorectomy was performed due to severe anemia from ongoing heavy menstrual bleeding, which was unresponsive to polypectomy. The patient was perimenopausal with no future fertility needs, justifying definitive surgery. There were no symptoms directly attributable to OSCC, possibly because the malignancy was asymptomatic or masked by overlapping adenomyosis-related symptoms, highlighting the risk of missing potentially fatal diagnoses like OSCC. This incidental finding significantly altered the management plan, necessitating chemotherapy, which would not be indicated for benign conditions such as adenomyosis or endometriosis. It also markedly changed the prognosis and long-term expectations for the patient.

No standardized treatment guidelines exist for OSCC. Management typically follows protocols for epithelial ovarian cancer, with debulking surgery including bilateral salpingo-oophorectomy with or without hysterectomy and staging procedures such as omentectomy and lymphadenectomy, followed by adjuvant carboplatin and paclitaxel chemotherapy [3-10]. In cases where the tumor is adherent to surrounding structures, resection of the appendix, colon, bladder, kidneys, ureters, omentum, or pelvic sidewall may be necessary. Fertility-sparing surgery may be considered in selected young patients desiring future fertility [4]. Peritoneal biopsies for accurate staging are widely recommended. The benefit of lymphadenectomy remains debated, with some studies showing improved prognosis [6]. Minimally invasive surgery offers faster recovery, but it risks tumor spillage and recurrence, so thorough peritoneal washing is recommended to reduce the risk of additional peritoneal lesions [6]. Recent studies suggest it may be safe if the specimen is secured in an endoscopic retrieval bag [6]. The first-line chemotherapy regimen is paclitaxel combined with platinum, such as carboplatin, although treatment responses vary and more data are needed to evaluate regimen efficacy [3-5]. Our patient also received this adjuvant chemotherapy following the unexpected diagnosis of OSCC. While squamous cell carcinomas are generally radiosensitive, current reports do not support the benefit of radiotherapy in OSCC; future research is warranted to explore the role of concurrent chemoradiation [8].

OSCC is an aggressive tumor with a poor prognosis, often associated with early recurrence or metastasis. Stage and age are important prognostic indicators [3-10]. The median overall survival is 42 months, with five-year cancer-specific survival rates of 86% for FIGO stage I and 54.3%, 36.3%, and 2.8% for stages II, III, and IV, respectively [4]. The prognosis of OSCC is generally worse than that of other epithelial ovarian cancers, including high-grade serous adenocarcinoma [3]. Cases of OSCC arising from endometriosis tend to have poorer survival than those originating from teratomas [4], although large-scale survival data specific to endometriosis-associated OSCC remain lacking. In a review by Zhang et al., among 42 OSCC patients, 21.4% had persistent or progressive disease, and 33.3% experienced recurrence on follow-up [4]. Poor outcomes are likely due to the insidious and nonspecific presentation, which delays diagnosis. In our case, the presence of symptomatic adenomyosis led to early surgical intervention and incidental detection of OSCC. The patient's postoperative course has been favorable so far, but the high proliferation index necessitates close ongoing surveillance.

## Conclusions

This case underscores the need to maintain a high index of suspicion for ovarian malignancy in women with adenomyosis and AUB, especially when adnexal abnormalities are present, as seen in our case, where the adenomyosis-related AUB masked symptoms of OSCC. Thorough histopathological evaluation, with extensive sampling of both ovarian lesions and adjacent endometriotic foci, is essential, as the rapidly growing squamous tumor often overgrows its endometriotic origin and obliterates diagnostic evidence. Determining the tumor’s origin is critical, since prognosis varies by histological subtype. Primary OSCC arising in endometriosis is exceedingly rare and aggressive. Its nonspecific presentation can mimic other disorders, leading to delayed diagnosis. Therefore, suspicion for malignancy should be high in all older women presenting with vague abdominal pain, distension, or palpable adnexal masses. Comprehensive imaging and prompt surgical management (debulking surgery with or without hysterectomy, omentectomy, and lymphadenectomy) followed by platinum‑based chemotherapy remain the mainstay of treatment, mirroring protocols for epithelial ovarian cancer, although no standardized guidelines exist. Multidisciplinary collaboration through tumor boards and registry reporting is vital to improve understanding of prognosis and to develop consensus‑based protocols. Our report adds to the limited literature on this rare histotype and may help establish future guidelines for optimal surgical intervention and adjuvant chemotherapy regimens.
